# Local Repressor AcrR Regulates AcrAB Efflux Pump Required for Biofilm Formation and Virulence in *Acinetobacter nosocomialis*

**DOI:** 10.3389/fcimb.2018.00270

**Published:** 2018-08-07

**Authors:** Bindu Subhadra, Jaeseok Kim, Dong Ho Kim, Kyungho Woo, Man Hwan Oh, Chul Hee Choi

**Affiliations:** ^1^Department of Microbiology and Medical Science, Chungnam National University School of Medicine, Daejeon, South Korea; ^2^Department of Nanobiomedical Science, Dankook University, Cheonan, South Korea

**Keywords:** *A. nosocomialis*, efflux pump, AcrR, antibiotic resistance, quorum sensing, biofilm, virulence

## Abstract

Multidrug efflux systems contribute to antimicrobial resistance and pathogenicity in bacteria. Here, we report the identification and characterization of a transcriptional regulator AcrR controlling the yet uncharacterized multidrug efflux pump, AcrAB in *Acinetobacter nosocomialis. In silico* analysis revealed that the homologs of AcrR and AcrAB are reported in the genomes of many other bacterial species. We confirmed that the genes encoding the AcrAB efflux pump, *acrA* and *acrB* forms a polycistronic operon which is under the control of *acrR* gene upstream of *acrA*. Bioinformatic analysis indicated the presence of AcrR binding motif in the promoter region of *acrAB* operon and the specific binding of AcrR was confirmed by electrophoretic mobility shift assay (EMSA). The EMSA data showed that AcrR binds to −89 bp upstream of the start codon of *acrA*. The mRNA expression analysis depicted that the expression of *acrA* and *acrB* genes are elevated in the deletion mutant compared to that in the wild type confirming that AcrR acts as a repressor of *acrAB* operon in *A. nosocomialis*. The deletion of *acrR* resulted in increased motility, biofilm/pellicle formation and invasion in *A. nosocomialis*. We further analyzed the role of AcrR in *A. nosocomialis* pathogenesis *in vivo* using murine model and it was shown that *acrR* mutant is highly virulent inducing severe infection in mouse leading to host death. In addition, the intracellular survival rate of *acrR* mutant was higher compared to that of wild type. Our data demonstrates that AcrR functions as an important regulator of AcrAB efflux pump and is associated with several phenotypes such as motility, biofilm/pellicle formation and pathogenesis in *A. nosocomialis*.

## Introduction

*Acinetobacter* spp. are important opportunistic bacterial pathogens primarily associated with nosocomial infections worldwide. These are the causative organisms for a number of pathological conditions including bacteremia, pneumonia, meningitis, urinary tract infections, and wound infections (Peleg et al., 2008; Lee et al., 2011; Nemec et al., 2011). The medically relevant *Acinetobacter* spp. predominantly come from a single group, designated as *Acinetobacter baumannii* (*Ab*) group which includes *A. baumannii, Acinetobacter nosocomialis, Acinetobacter pittii, Acinetobacter seifertii*, and *Acinetobacter dijkshoorniae*. All the members of the *Ab* group are opportunistic pathogens and have the capacity to acquire multidrug resistance phenotype (Nemec et al., 2015; Cosgaya et al., 2016; Marí-Almirall et al., 2017). *A. nosocomialis* is the most comprehensively studied strain in the *Ab* group second to *A. baumannii. A. baumannii* is reported to rapidly develop resistance to multiple antibiotics and is one of the most challenging pathogens for healthcare institutions (Fournier and Richet, 2006; Antunes et al., 2014; Doi et al., 2015). The increasing rate of multidrug resistance (MDR) phenotypes associated with *Acinetobacter* infections are so alarming that the Centers for Disease Control and Prevention (CDC) has listed *Acinetobacter* as a severe threat to mankind and last line therapeutic options like carbapenems, tigecycline, and colistin are now recommended to treat majority of *Acinetobacter* infections (Peleg and Hooper, 2010; CDC, 2013). Moreover, according to the recent epidemiological data, MDR strains acquire multidrug resistance phenotype faster than any of the other Gram-negative bacteria reported over the last decade (Giammanco et al., 2017).

Multidrug efflux pumps contribute to antibiotic resistance by extruding out drugs and are found in almost all bacterial species. These pumps have been categorized into five families on the basis of sequence similarity: resistance-nodulation-cell division (RND), the major facilitator, small multidrug resistance, multidrug and toxic compound extrusion, and ATP-binding cassette (ABC) families (Putman et al., 2000; Paulsen et al., 2001). In Gram negative bacteria, RND family efflux pumps are mostly effective in resistance to antibiotics (Nikaido, 1996; Murakami et al., 2002, 2006) and consist of an RND protein (the inner membrane component), membrane fusion protein (MFP: the periplasmic component), and the outer membrane protein (OMP: the outer membrane protein). The RND pumps were first reported in *Escherichia coli* and *Pseudomonas aeruginosa* independently and those include AcrAB-TolC (*E. coli*) (Ma et al., 1993, 1995), MexAB-OprM, MexCD-OprJ, MexEF-OprN etc. (*P. aeruginosa*) (Poole et al., 1993, 1996; Gotoh et al., 1995; Köhler et al., 1997). In *E. coli*, the increased level of antibiotic resistance is attributed majorly by this housekeeping efflux system AcrAB-TolC (Thanassi et al., 1997). The substrate profile of the AcrAB-TolC efflux pump in *E. coli* includes chloramphenicol, fluoroquinolone, tetracycline, novobiocin, rifampin, fusidic acid, nalidixic acid, and β-lactam antibiotics (Piddock, 2006). Apart from *E. coli*, the function of AcrAB efflux pump or its homologs on multidrug resistance has been reported in a number of bacterial strains including pseudomonads, *Salmonella enterica* serovar Typhimurium, *Enterobacter aerogenes, Klebsiella pneumoniae, Enterobacter cloacae, Campylobacter* spp., and *Borrelia burgdorferi*, etc. (Poole et al., 1993; Pumbwe et al., 2004; Buckley et al., 2006; Bunikis et al., 2008). Like that in *E. coli*, the AcrAB-TolC system in *S. enterica* is reported to actively expel different classes of antimicrobial agents such as quinolones, chloramphenicol, tetracycline, and nalidixic acid (Poole, 2007; Nishino et al., 2009).

The efflux pumps are almost exclusive to *A. baumannii, A. pittii*, and *A. nosocomialis* and their orthologues are distributed in other bacterial pathogens, such as *Pseudomonas* spp. The AdeABC efflux pump of *A. baumannii* confers resistance to various classes of antibiotics including aminoglycosides, tetracyclines, erythromycin, chloramphenicol, trimethoprim, fluoroquinolones, some β-lactams, tigecycline etc. (Magnet et al., 2001; Marchand et al., 2004).

The *adeABC* operon is found in 80% of the clinical isolates and its expression is tightly regulated by the two-component system, AdeRS (Marchand et al., 2004). A second RND efflux pump was reported in *A. baumannii* known as the AdeIJK which contributes resistance to β-lactams, chloramphenicol, tetracycline, tigecycline, erythromycin, lincosamides, fluoroquinolones, fusidic acid, novobiocin, rifampin, trimethoprim, acridine, safranin, pyronine, and sodium dodecyl sulfate (Laurence et al., 2007). The RND efflux pump, AdeFGH, encoded by *adeFGH* operon provides high-level resistance to fluoroquinolones, chloramphenicol, trimethoprim, clindamycin etc. and is under the control of a putative LysR-type transcriptional regulator, named *adeL*, which is located upstream of the *adeFGH* operon (Coyne et al., 2010a,b). Besides their role in antibiotic resistance, RND efflux pumps contribute to virulence and biofilm formation in *A. baumannii* (Richmond et al., 2016).

AnoR is a LuxR-type transcriptional regulator controlling the production of quorum sensing signals, *N*-acyl homoserine lactones (AHLs) in *A. nosocomialis* ATCC 17903 (Oh and Choi, 2015). Recently, we analyzed the whole transcriptome of *A. nosocomialis* wild-type (WT) and the *anoR* deletion mutant (unpublished data), and it revealed the downregulation of two uncharacterized efflux pump genes, NCTC8102_00280 and NCTC8102_00290. *In silico* analysis pointed out that these genes belong to the RND-type transport system and many homologs are found in the bacterial community. The NCTC8102_00280 and NCTC8102_00290 showed identity to the *acrA* and *acrB* genes of the *acrAB* operon, constituting the AcrAB-TolC efflux pump (Okusu et al., 1996) of *E. coli*. The regulation of expression of RND pumps are tightly regulated by an intricate balance between local regulators (generally repressors) and global regulators. It has been reported previously that *acrAB* is under the control of a local repressor, AcrR in *E. coli* (Ma et al., 1996). Further *in silico* analysis of the *A. nosocomialis* genome revealed an AcrR homolog upstream of *acrA* of the *acrAB* operon. In this study, we report the regulation of *acrAB* efflux pump by AcrR and the data indicate that AcrR plays a major role in motility, biofilm/pellicle formation and pathogenesis in *A. nosocomialis* by repressing the *acrAB* operon.

## Materials and methods

### Bacterial strains, plasmids, and culture conditions

Bacterial strains and plasmids used in this study are shown in Table 1. Unless otherwise stated, *E. coli* strains were grown in Luria-Bertani (LB) medium (10 g/L tryptone, 5 g/L yeast extract, 10 g/L NaCl) at 37°C, while the *A. nosocomialis* strains were grown at 30 or 37°C in either LB or Mueller Hinton (MH) media (Difco). When appropriate, antibiotics were added to media for *E. coli* (kanamycin, 50 μg/ml; chloramphenicol, 20 μg/ml) as well as *A. nosocomialis* (kanamycin, 30 μg/ml; ampicillin, 100 μg/ml). Human aveolar epithelial cell line (A549) was cultured in Dulbecco's Modified Eagle's Medium (DMEM, WELGENE Inc., Korea) supplemented with 10% fetal bovine serum (FBS, WELGENE Inc., Korea) and penicillin G (100 U/ml)/streptomycin (100 ug/ml). The cells were maintained at 37°C in 5% (vol/vol) CO_2_ in 75-cm^2^ tissue culture flasks until used.

**Table 1:** Bacterial strains and plasmids used in this study.

**Strain/Plasmid**	**Relevant characteristics^a^**	**References**
***Acinetobacter nosocomialis*** **STRAINS**
ATCC 17903	Type strain	ATCC
Δ*acrR*	*acrR* deletion mutant of ATCC 17903	This study
***Escherichia coli*** **STRAINS**
DH5α	F- *thi-I end A1 hsdR17* (r^−^m^−^) *supE44* Δ*lacU169* (Φ80*lacZ*ΔM15) *recA1 gyrA96 relA*	Hanahan, 1983
DH5α λ *pir*	*supE44 ΔlacU169*(Φ80 *lacZ*ΔM15) *hsdR17 recA1 endA1 gyrA96 thi-1 relA1 λpir* (phage lysogen); plasmid replication	Laboratory collection
S17-1 λ *pir*	*λpir* lysogen; *thi pro hsdR hsdM*^+^ *recA* RP4-2 Tc::Mu-Km::Tn7; Tp^r^ Sm^r^; host for π-requiring plasmids; conjugal donor	Simon et al., 1983
BL21(DE3)	*ompT hsdS*_B_(${\text{r}}_{\text{B}}^{-}$${\text{m}}_{\text{B}}^{-}$) *gal dcm* (DE3)	Studier and Moffatt, 1986
**PLASMIDS**
pUC4K	pUC4 with *nptI*; Ap^r^, Km^r^	Pharmacia
pHKD01	pDS132, multicloning sites; *ori*R6K *sacB*; Cm^r^	Oh et al., 2015
pOH959	pHKD01 with Δ*acrR*::*nptI*; Cm^r^, Km^r^	This study
pOH993	pHKD01 with the coding region of *acrR* under control of its native promoter with *nptI*; Cm^r^, Km^r^	This study
pET-*acrR*	pET_28a carrying the coding region of *acrR*	This study

a *Tp^r^, trimethoprim resistant; Sm^r^, streptomycin resistant; Ap^r^, ampicillin resistant; Km^r^, kanamycin resistant; Cm^r^, chloramphenicol resistant*.

### DNA manipulations

Standard cloning procedures were followed in this study (Sambrook et al., 1989). Oligonucleotides were purchased from Macrogen Co., Ltd., Korea and all the oligonucleotides used in this study are given in Table 2. The genomic DNA from *A. nosocomialis* and the plasmid DNA from *E. coli* were isolated using the PureHelix™ Genomic DNA Prep Kit (Nanohelix Co. Ltd., Korea) and AccuPrep® Plasmid Extraction Kit (Bioneer Corp., Korea), respectively. The plasmids were introduced into *E. coli* and *A. nosocomialis* by heat shock method (Hanahan, 1983) and conjugation (Oh et al., 2015), respectively. PrimeSTAR GXL Taq DNA polymerase (TaKaRa, Japan) was used for polymerase chain reaction (PCR) amplification of DNA fragments. The DNA fragments were purified or eluted from gel using HiGene™ Gel & PCR Purification System (Biofact Co., Ltd., Korea). Restriction and DNA modifying enzymes were purchased from New England Biolabs, USA. The nucleotide sequence of all recombinant strains and mutants were confirmed by sequencing (Macrogen Co., Ltd., Korea).

**Table 2:** Oligonucleotides used in this study.

**Oligonucleotides**	**Sequence (5′ → 3′)^a,b^**	**Purpose**
AcrR01F	AAAACCCTCTAATAAAAGATTAAAAAAATAA	Construction of *acrR* deletion mutant
AcrR01R	ACCTTCTTCACGAGGCAGACCATAGGGCTGATTATCTGTGGC	Construction of *acrR* deletion mutant
AcrR02F	CAAGCCTGAATTGAAAGCTCC	Construction of *acrR* deletion mutant
AcrR02R	ATCTTTTATTAGAGGGTTTTACCAAAGTCCGATTGCCAAA	Construction of *acrR* deletion mutant
AcrR03F	TTGTGATAAATAAAATTTATGCTATTTTCTCCTGTTCTTATTCTTATC	Complementation of *acrR* deletion mutant
AcrR03R	ATTAATTTAGTCATAAAAACATCTTATCGCTCTTATTCAAACTGAT	Complementation of *acrR* deletion mutant
PhaC01F	AAAGATCAAAGGCACCACAATG	Complementation of *acrR* deletion mutant
PhaC01R	ATAAATTTTATTTATCACAAAACCATTTT	Complementation of *acrR* deletion mutant
PhaC02F	GTTTTTATGACTAAATTAATATATTGGTTAACT	Complementation of *acrR* deletion mutant
PhaC02R	ACCTTCTTCACGAGGCAGACCCTACACGCAGCCTTATACGTT	Complementation of *acrR* deletion mutant
U1	GTCTGCCTCGTGAAGAAGGTG	Amplification of *nptI*
U2	GATCCGTCGACCTGCAGG	Amplification of *nptI*
280-290_F	GCAGAAGATCCATCCGATTG	Confirmation of *acrAB* as polycistronic operon
280-290_R	TGTAACCATCGACTCACCTG	Confirmation of *acrAB* as polycistronic operon
PET_*acrR*_F	GGAATTC*CATATG*GTGCAAACAGTTAATCAAAG	For overexpression of AcrR
PET_*acrR*_R	CCG*CTCGAG*CGGATTCCGATATTTGAGGAAAAA	For overexpression of AcrR
P*acrA*_F	AAAACCCTCTAATAAAAGATTAA	For EMSA
P*acrA*_R	ATCTTATCGCTCTTATTCAAA	For EMSA
16s rDNA_F	CGTGCTACAATGGTCGGT	For EMSA
16s rDNA_R	GTATTCACCGCGGCATTC	For EMSA
16S_F	AAGACTAAAACTCAAATGAA	For qRT-pCR
16S_R	TGGAAAGTTCTTACTATGTC	For qRT-pCR
AcrA_F	GATGGTAACGCAGCCTTC	For qRT-pCR
AcrA_R	GCCGTAACTTGTCCACCT	For qRT-pCR
AcrB_F	AGCGACTGTTGTTGGCGA	For qRT-pCR
AcrB_R	TACCAGCATTGACCG AAC	For qRT-pCR
*csuC*_F	GTCAGTCCGCAACAATGACA	For qRT-pCR
*csuC*_R	ACAACACATAGCCGAAAGCA	For qRT-pCR
*csuD*_F	ACTGTTCCAACGAAACGCAA	For qRT-pCR
*csuD*_R	CGTCGGGTTAAATCGACACC	For qRT-pCR

a *Regions of oligonucleotides that are not complementary to the corresponding templates are underlined*.

b *Added restriction site sequences are indicated in italics*.

### *In silico* analysis

*In silico* analysis was performed to identify homologs of *acrAB* operon and *acrR*, using the *A. nosocomialis* ATCC 17903 (NCTC 8102) genome sequence retrieved from GenBank (GenBank accession no. CP029351). Phylogenetic analysis and molecular evolutionary analyzes were conducted to determine the relationship of *acrR* of *A. nosocomialis* with that of other related strains. The strains used for the construction of phylogenetic tree are given in Supplementary Table 1. Phylogenetic tree was constructed using MEGA 6.06 (Tamura et al., 2013). The sequences were aligned using MUSCLE (Edgar, 2004) and pairwise distances were estimated using the Maximum Likelihood approach. In order to find out the binding motif of AcrR in the promoter region of *acrA*/*acrR* (P*acrA*/*acrR*) of *A. nosocomialis*, multiple sequence alignment was carried out with the P*acrA*/*acrR* and the known AcrR binding motif from *E. coli* (Su et al., 2007).

### Overexpression and purification of His_6_-AcrR

For the overexpression of hexahistidyl-tagged AcrR (His_6_-AcrR), the complete coding region of *acrR* (624 bp) of *A. nosocomialis* was amplified by PCR and subcloned into His-tag expression vector, pET28a yielding pET-*acrR*. The recombinant plasmid was introduced into *E. coli* strain BL21(DE3) by transformation. The AcrR was overexpressed by adding 1 mM isopropyl β-D-1-thiogalactopyranoside (IPTG) at an OD_600_ of 0.5 and incubated the culture further for 5 h at 30°C. Induced cells were harvested by centrifugation and lysed by sonication (Vibra-Cell™, Sonics & Materials, Inc., USA) in lysis buffer [20 mM Tris-HCl (pH 8), 500 mM NaCl and 5 mM imidazole]. After centrifugation, the soluble supernatant was mixed with nickel–nitrilotriacetic acid–agarose solution (Qiagen, Germany) and loaded into polypropylene column (Thermo Fisher Scientific, USA). The column was washed with lysis buffer and the protein was eluted in elution buffer with varying imidazole concentration [20 mM Tris-HCl (pH 8), 500 mM NaCl, and 150/300 mM imidazole]. The purified AcrR was dialyzed overnight against phosphate buffered saline (PBS) to remove imidazole and directly used for the promoter binding assay.

### Electrophoretic mobility shift assay (EMSA)

EMSA was carried out to check the binding of AcrR to the promoter region of *acrA*/*acrR* according to the protocol described earlier (Alves and Cunha, 2012). A promoter fragment of 148 bp and 16s rDNA of 149 was amplified by PCR using the oligonucleotide primers listed in Table 2. In the binding assay, 200 ng of the P*acrA*/*acrR* fragment was incubated with varying amounts of purified His_6_-AcrR (0 to 0.5 μg) in 15 μl of binding buffer [250 mM phosphate buffer (pH 7.5), containing 50 mM NaCl, 500 mM KCl, 10 mM dithiothrietol, 10 mM ethylenediaminetetraacetic acid, 3 μg bovine serum albumin and 1 μg Poly[d(I-C)]]. To confirm the binding specificity of AcrR, 16s rDNA was included in the EMSA reaction mixture instead of the promoter fragment. The binding assay was carried out at room temperature (RT) for 30 min and the reaction mixtures were electrophoresed in 6% prerunned (20 min at 80 V) native polyacrylamide gel in 1x Tris/Borate/EDTA (TBE) buffer for 1 h at 80 V followed by staining with ECOred (MOSAICON, Korea) and visualization.

### RNA extraction and real-time quantitative reverse transcription PCR (RT-PCR)

Cultures were inoculated into LB medium and incubated until the exponential phase. Total RNA was extracted with High Pure RNA Isolation Kit (Roche Diagnostics GmbH, Germany) according to the manufacturer's instructions. Complementary DNA (cDNA) was synthesized from 1 μg of DNase-treated total RNA with Reverse Transcription Master Premix (ELPIS Biotech. Inc., Korea) per manufacturer's instructions. Quantitative RT-PCR was performed in triplicates using StepOnePlus™ Real-Time PCR System (Applied Biosystems, USA) with Power SYBR® Green PCR Master Mix (Applied Biosystems, USA) and indicated primers (Table 2). Gene expression was normalized by the ΔΔCT method (Livak and Schmittgen, 2001) and 16S rRNA was used as a reference.

### Construction of *acrR* deletion mutant and complemented strain

To construct the deletion mutant of *acrR*, markerless gene deletion method was followed as described previously (Oh et al., 2015). The complete open reading frame of the *acrR* (624 bp) was deleted by overlap extension PCR. For this, the upstream and downstream regions of the *acrR* were amplified using two sets of primer pair (AcrR01F and AcrR01R; AcrR02F and AcrR02R). *nptI* conferring kanamycin resistance was amplified from pUC4K using the primers, U1 and U2. Overlap extension PCR was carried out to anneal the three PCR products at their overlapping regions using the primers AcrR01F and U2. The fusion product was ligated into FspI-digested pHKD01 to result in pOH959. The recombinant plasmid pOH959 was introduced into *A. nosocomialis* by conjugation as described previously (Oh et al., 2015) and the integration of POH959 into the chromosome was confirmed by selection of colonies on LB plate containing ampicillin and kanamycin. To screen the colonies having mutated *acrR* gene, kanamycin-resistant colonies were grown overnight in LB plate containing 10% (w/v) sucrose. A sucrose-resistant and kanamycin sensitive cell (*acrR* deletion mutant) was selected and the double crossover event was confirmed by PCR. For single-copy *acrR* complementation in the *acrR* mutant, the *acrR* including its native promoter was inserted into the intergenic region that is located downstream of *phaC* encoding alpha/beta hydrolase by a modified markerless gene deletion method. Briefly, the *acrR* including its promoter region and the downstream regions (part I and part II) of *phaC* were amplified using the primer pairs AcrR03F/AcrR03R, PhaC01F/PhaC01R, and PhaC02F/PhaC02R, respectively. The *nptI* was amplified from the pUC4K plasmid using the primer pair U1/U2. The primers, AcrR03F and AcrR03R contained additional 20 nucleotides at their 5′ ends that are complementary to the regions of part I and part II of the downstream region of *phaC*, respectively, while PhaC02R carried additional 20 nucleotides at its 5′ end that is complementary to the part of kanamycin resistance cassette. All the four PCR products obtained were annealed together by overlap extension PCR using the primers PhaC01F and U2. The recombinant DNA fragment from the overlap extension PCR was ligated into the FspI-digested pHKD01 to generate pOH993. The recombinant plasmid, pOH993 was introduced into *acrR* deletion mutant by conjugation and the recombinants were screened as described previously (Oh et al., 2015).

### Motility test

Surface-associated motility was examined on MH plate containing 0.25% Eiken soft agar (Eiken chemical, Co., Ltd., Japan) as described previously (Skiebe et al., 2012). In order to minimize the variation between plates, 30 ml of the motility agar was poured to each plate in the laminar flow hood and the plates were dried for 30 min. *A. nosocomialis* WT, *acrR* deletion mutant and complemented mutant strains cultivated in MH broth overnight were diluted to an OD_600_ of 1 and 5 μl of the samples were spotted onto the motility plate. The plates were incubated at 30°C for 18 h and the motility on top of agar was observed. The plates were prepared fresh for each surface motility experiment and the test was repeated on at least three separate occasions.

### Biofilm/pellicle formation assay

Biofilm and pellicle formation assays were adapted from previously described protocols with slight modifications (Lee et al., 2008; Giles et al., 2015). For biofilm and pellicle formation assay, *A. nosocomialis* WT, *acrR* deletion mutant and complemented strains were cultivated in MH broth at 37°C. The overnight cultures were diluted to an OD_600_ of 1 and 25 μl of the diluted cultures were added to 5 ml of MH broth in polystyrene tubes (Ø 17 mm × H 100 mm) (SPL Life Sciences, Korea). The tubes were incubated at different time periods at 30°C without shaking. For biofilm assay, planktonic cells were removed post-incubation and washed thrice with sterile distilled water. For pellicle assay, 100% ethanol was carefully added to the bottom of the pellicle and the pellicle was removed and resuspended in 1 ml of PBS. The pellicle suspension was centrifuged at 13,000 rpm and the supernatant was carefully removed. Both biofilm and pellicle was stained with 0.1% (wt/vol) crystal violet (Sigma, USA) for 15 min at RT followed by washing with sterile distilled water thrice and air drying at RT. The stain absorbed to biofilm or pellicle was eluted with 30% acetic acid and quantitated by measuring the optical density at 595 nm. The pellicle/biofilm assay was performed in quintuplicates at three different occasions.

### Adherence assay

To check the adherence ability of *acrR* deletion mutant, A549 epithelial cells were seeded on 13 mm cell culture coverslips (Nunc™, Thermo Scientific, USA) in 24-well plates containing DMEM (WELGENE Inc., Korea) supplemented with 10% FBS (WELGENE Inc., Korea) and incubated at 37°C in 5% (vol/vol) CO_2_ for 24 h. *A. nosocomialis* WT, *acrR* deletion mutant and complemented strains were grown in LB broth overnight at 37°C, washed and diluted in DMEM containing FBS. The cell line was infected with different *A. nosocomialis* strains at a ratio of bacteria to host cells of 100:1 (MOI of 100) and incubated at 37°C in 5% CO_2_ for 5 h. After washing five times with Dulbecco's Phosphate Buffered Saline (DPBS), the cells were fixed with methanol for 20 min, and stained with Giemsa solution (Lee et al., 2006).

### Invasion assay

For the invasion assay, 1 × 10^5^ A549 epithelial cells were seeded into 24-well plates containing DMEM (WELGENE Inc., Korea) supplemented with 10% FBS (WELGENE Inc., Korea) and incubated at 37°C in 5% (vol/vol) CO_2_ for 24 h. Cultures of *A. nosocomialis* WT, *acrR* deletion mutant and complemented strains were grown in LB broth overnight at 37°C, washed and diluted in DMEM supplemented with FBS. The cell line was washed with DPBS (WELGENE Inc., Korea) and infected with the bacterial cultures at an MOI of 100. After incubation for 5 h at 37°C in 5% (vol/vol) CO_2_, the wells were washed three times with DPBS before adding DMEM-FBS containing 300 μg/ml of gentamycin to kill all external bacteria. The microplates were incubated at 37°C in 5% CO_2_ for 2 h. The cells were washed with DPBS, lysed with Ultrapure water (WELGENE Inc., Korea) containing 0.25% Triton X-100 for 20 min at RT and appropriate dilutions were dropped onto LB agar plates. The plates were incubated overnight and CFUs were counted to quantify bacteria surviving intracellularly. To visualize the invasion of bacteria by fluorescence microscopy, cells were seeded on 13 mm glass coverslips and incubated for 24 h. After infection for 5 h, the cells were fixed with 3.7% paraformaldehyde for 10 min and permeabilized with 0.25% Triton X-100. Bacterial cells were labeled with polyclonal anti-rabbit AbOmpA antibody (1:1,000) followed by Alexa Fluor® 594-conjugated goat anti-rabbit IgG antibody (Invitrogen, USA). Actin was stained with Alexa Fluor® 488 phalloidin (Invitrogen, USA) and nucleus with DAPI (6-diamidino-2-phenylindole, Invitrogen, USA). The invasion of *A. nosocomialis* strains into epithelial cells was observed under Leica DMi8 confocal microscope (Leica Microsystems Ltd. Germany).

### Animal experiments

Seven weeks old female BALB/c mice were used to assess the virulence of the *acrR* deletion mutant. The mice were obtained from Nara Biotech, Korea and the animals were maintained under specific-pathogen-free conditions according to the Institutional Animal Care and Use Committee (IACUC) of Chungnam National University guidelines (permission number: CNU-00999). The animal experiments were performed in accordance with the guidelines of Korean Food and Drug Administration. For induction of neutropenic mouse model, 7-week-old female BALB/c mice were injected intraperitoneally with 100 mg of cyclophosphamide (CTX, Sigma. USA)/kg of body weight on day 1 and 4 prior to injection of bacterial cells, respectively. The mice were anesthetized and groups containing three were infected intratracheally with 1 × 10^8^ CFU/ml of *A. nosocomialis* WT, *acrR* deletion mutant and complemented mutant. One hundred microliters of PBS was used to inject the control mice group. Mice were sacrificed 3 days post-infection, and lungs were harvested to assess the bacterial load. The lungs were homogenized and the cell suspension was serially diluted and spotted onto LB plates to count the bacteria. For histological analysis, tissues were subjected to hematoxylin/eosin, or immunohistochemical staining with OmpA (outer membrane protein A) antibody to visualize the bacteria.

### Statistical analysis

Whenever applicable, the experiments were repeated at least three times with consistent results. The significance of difference between two groups was determined by unpaired Student's *t*-test and that among more than three groups was evaluated with one-way ANOVA followed by Tukey's multiple comparison test using statistical software GraphPad Prism v5.01 (GraphPad Software Inc., USA). ^*^P < 0.05, ^**^P < 0.01, and ^***^P < 0.001 were considered as statistically significant.

## Results

### The *A. nosocomialis* genome encodes *acrAB* operon which is under the control of *acrR*

**Figure 1:**
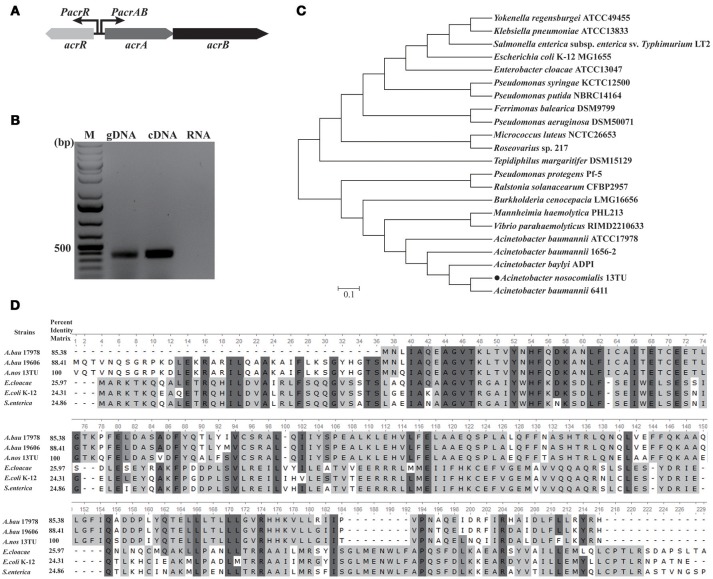
Genomic organization of *acrAB* operon and *in silico* analysis of *acrR*. **(A)** The schematic representation of the *acrAB* efflux pump operon in *A. nosocomialis*. The *acrR* gene which is divergently transcribed from the *acrAB* operon shares a common promoter region with the *acrAB* operon. **(B)** Confirmation of *acrAB* as polycistronic operon. RT-PCR analysis was performed using total RNA isolated from *A. nosocomialis* wild type strain grown in LB medium at 37°C. A negative control and positive control reactions were performed using DNase-treated RNA and genomic DNA as templates, respectively. The size of the amplified fragments was estimated by comparing it with the size marker DNAs (lane M). **(C)** Phylogenetic analysis of *acrR* from *A. nosocomialis* with that of other bacterial strains. The tree was constructed using MEGA 6.06 and the pairwise distance was calculated using Maximum Likelihood approach according to the aligned sequences. **(D)** Multiple sequence alignment of AcrR from *A. nosocomialis* with homologs from different bacterial strains. Residues that are conserved across all sequences are highlighted in dark gray and matches across the sequences by light gray. Pairwise sequence identity (%) is also shown. *A.bau* 17978, *A. baumannii* 17978; *A.nos* 13TU, *A. nosocomialis*; *A.bau* 19606; *A. baumannii* 19606.

In this study, *in silico* analysis revealed the presence of a well-defined *acrAB* efflux pump operon in *A. nosocomialis* comprising of *acrA*, which encodes the membrane protein, and *acrB*, encoding acriflavine resistance protein (Figure 1A). The *acrA* and *acrB* genes of *A. nosocomialis* showed 24 and 25% identity to those of *E. coli*, respectively. The gene which encodes the outer membrane protein of the AcrAB efflux pump of *A. nosocomialis* is yet to the identified. The *acrAB* efflux pump is under the control of *acrR* gene upstream of it (Figure 1A) and the *acrAB* operon and *acrR* are divergently transcribed under the same promoter activity. We confirmed that *acrAB* is a polycistronic operon by amplifying the genomic region covering the 3′-end of *acrA* and 5′-end of *acrB* using cDNA prepared from DNAse-treated RNA (Figure 1B). The *acrR* is widely distributed among bacterial species and phylogenetic analysis was carried out to find out the relationships of *acrR*. The *acrR* from *Acinetobacter* strains showed close similarity with that from *V. parahaemolyticus* RIMD 2210633 and *M. haemolytica* PHL213 (Figure 1C). Also, the AcrR from *A. nosocomialis* shared high similarity of 85.38% and 88.41% with that of *A. baumannii* 17978 and *A. baumannii* 19606, respectively (Figure 1D). The well-studied AcrR from *E. cloacae, E. coli* K12 and *S. enterica* shared similarity of 25.9, 24.31, and 24.86%, respectively.

### AcrR binds to the promoter region of *acrA/acrR*

**Figure 2:**
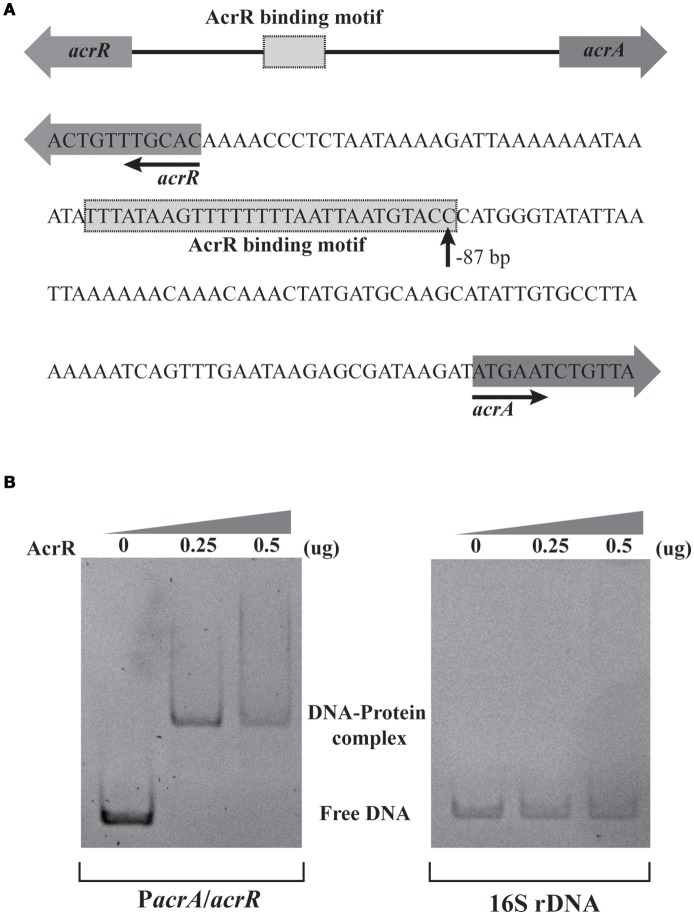
EMSA analysis for the AcrR interaction with the *acrR*-*acrAB* intergenic region. **(A)** Scheme and nucleotide sequence of the intergenic region of *acrR* and *acrAB*. A putative AcrR binding motif located in the promoter region between *acrR* and *acrA* is shown. The number beneath the indicated binding site of AcrR points the position of the first nucleotide in the binding motif relative to the translational start site of *acrA*. **(B)** EMSA result showing the interaction between AcrR and the promoter region of *acrA*/*acrR*. The promoter region and 16s rDNA (as control) was amplified by PCR and incubated without or with different concentrations of purified AcrR. 1 μg of poly[d(I-C)] was added to each reaction mixture in order to avoid non-specific binding.

In *E. coli*, AcrR functions as one of the regulators modulating the expression of *acrAB* operon (Ma et al., 1996). In this study, *in silico* analysis revealed the presence of an AcrR binding motif in the promoter region of *acrA*/*acrR* (Figure 2A). In order to examine the direct interaction between AcrR and P*acrA*/*acrR*, EMSA was performed with a 148 bp fragment of the promoter region and purified AcrR. With the addition of AcrR, the free DNA substrate disappeared and the DNA bands shifted (Figure 2B), indicating that AcrR could bind to the *acrAB* regulatory region. The observed interactions were specific because no AcrR binding of a 16S rDNA control fragment was observed (Figure 2B). The AcrR binding motif was located at position −87 (*TTTA*T*A*AGTT*TTT*T*T*T*AAT*TA*ATGTACC*) relative to the translational start site (Figure 2A), according to the consensus sequences reported earlier in *E. coli* (Su et al., 2007). The consensus bases matching with those in the AcrR binding motif in *E. coli* are underlined. The EMSA data clearly shows that AcrR specifically binds to the promoter region of *acrA*/*acrR* and thus suggests an involvement in the regulation of expression of *acrAB* operon.

### AcrR negatively regulates *acrAB* expression

**Figure 3:**
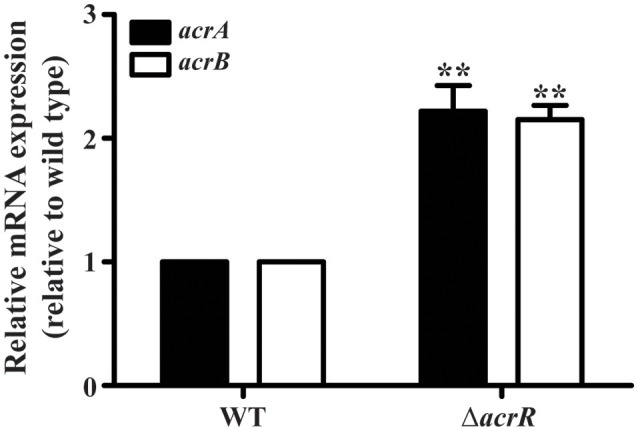
Transcription of *acrA* and *acrB* are significantly upregulated in the *acrR* deletion mutant. The relative *acrA* (black bars) and *acrB* (white bars) mRNA expression levels in the wild type (WT) and *acrR* mutant (Δ*acrR*) were determined as described in the section “Materials and Methods.” Values are mean ± SD (*n* = *3*) and asterisks indicate a significant difference in mRNA expression between the mutant and the wild type at **P < 0.01.

Previous studies have pointed out that the expression of *acrAB* operon is downregulated by the local repressor AcrR in *E. coli* and *S*. Typhimurium (Olliver et al., 2004; Pourahmad Jaktaji and Jazayeri, 2013). In *A. nosocomialis*, the DNA-binding assay revealed an interaction of AcrR with the promoter region of *acrA*/*acrR*. To functionally analyze the effect of AcrR on *acrAB* expression in *A. nosocomialis*, RT-PCR transcription analyses were performed using wild type and *acrR* mutant strains. Deletion of *acrR* increased *acrA* and *acrB* expression in the mutant compared to the wild type (Figure 3). The data demonstrates that AcrR negatively regulates the expression of *acrAB* operon, similar to that in *E. coli* and *S*. Typhimurium.

### Deleting *acrR* enhances motility

**Figure 4:**
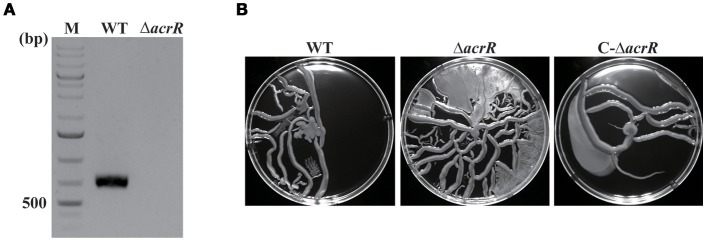
Confirmation of *acrR* deletion by PCR and motility assay. **(A)** Verification of *acrR* deletion mutant was carried out by PCR using genomic DNA as template. WT, wild type; Δ*acrR, acrR* mutant; M, DNA molecular weight marker. **(B)** Enhanced motility when *acrR* is deleted. Overnight cultures of *A. nosocomialis* wild type, *acrR* mutant and complemented mutant were cultivated in MH medium containing 0.25% Eiken agar and incubated at 30°C. The plates were observed after 18 h and photographed. The motility experiment was conducted in triplicates at three different occasions. Values are mean ± SD (*n* = *3*).

The *acrR* deletion mutant of *A. nosocomialis* ATCC 17903 was constructed using markerless gene deletion method based on double cross over. The detailed protocol for the construction of deletion mutant is given in the “Materials and Methods” section. The whole *acrR* gene was deleted and the deletion was confirmed by PCR (Figure 4A) followed by sequencing. For the complementation of *acrR* mutant, the *acrR* coding region along with its native promoter was inserted into intergenic region that is located downstream of *phaC* encoding alpha/beta hydrolase. The complementation of the *acrR* mutant was confirmed by PCR. To characterize the *acrR* mutant, *A. nosocomialis* wild type, *acrR* mutant and complemented mutant were cultivated in MH broth and the OD_600_ was measured until 24 h of incubation. It was noticed that the growth pattern of *acrR* mutant was not different from that of wild type.

It has been reported previously that AcrR modulates motility in *E. coli* (Kim et al., 2016). To check the effect of *acrR* deletion on motility in *A. nosocomialis*, overnight cultures of *A. nosocomialis* WT, *acrR* deletion mutant and complemented mutant were spotted on MH medium containing 0.25% Eiken soft agar. The data showed that motility of the *acrR* mutant is significantly increased compared to that of wild type, indicating that AcrR plays an important role in the regulation of motility in *A. nosocomialis* (Figure 4B). As expected, the *acrR* complemented strain restored the motility similar to that of the wild type strain.

### Deletion of *acrR* increases biofilm/pellicle formation

**Figure 5:**
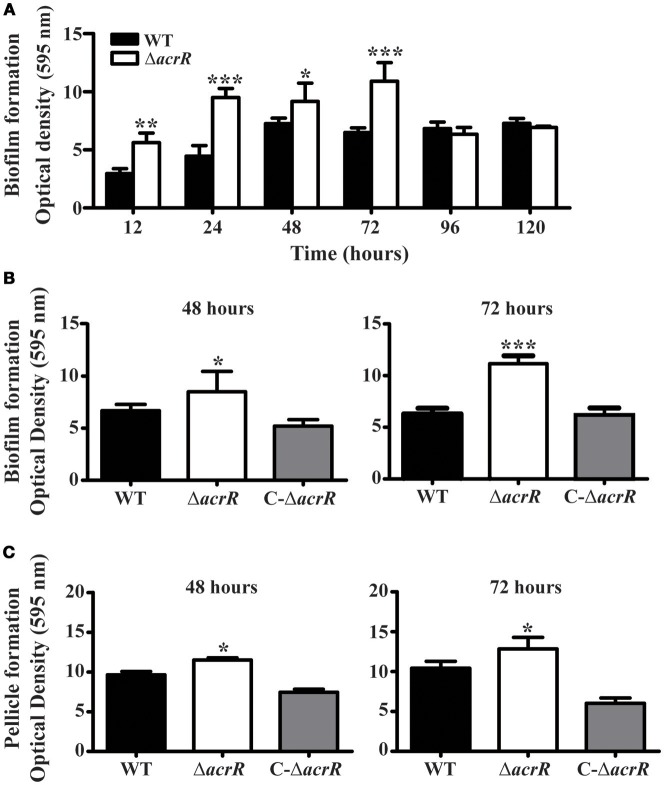
*acrR* mutant displays increased biofilm/pellicle formation. The strains were cultivated in MH medium at 30°C and biofilm/pellicle formation was quantified at different time periods based on crystal violet staining method. **(A)** Time-dependent biofilm formation by *A. nosocomialis* wild type and *acrR* mutant. **(B)** Crystal violet staining of the biofilm formation in wild type (WT), *acrR* mutant (Δ*acrR*) and complemented mutant (C-Δ*acrR*) at 48 and 72 h. Graphical representation of the biofilm formation is also shown. **(C)** The pellicle formation in wild type (WT), *acrR* mutant (Δ*acrR*) and complemented mutant (C-Δ*acrR*) at 48 h and 72 h are shown. Values are mean ± SD (*n* = *5*) and asterisks indicate significantly different average biofilm or pellicle value when compared with wild type at *P < 0.05, **P < 0.01, and ***P < 0.001. The biofilm/pellicle assay was performed at three different occasions.

Multidrug efflux pumps are known to be important constituent of biofilm formation (Lynch et al., 2007; Kvist et al., 2008; Matsumura et al., 2011; Baugh et al., 2012). In *S. enterica* serovar Typhimurium, the mutation in the *acrAB* operon led to impaired biofilm formation (Baugh et al., 2014; Schlisselberg et al., 2015). Similarly, the *acrB* mutant of *E. coli* also depicted decreased biofilm formation (Kvist et al., 2008; Matsumura et al., 2011). Therefore, we checked the effect of *acrR* deletion on biofilm/pellicle formation in *A. nosocomialis* by cultivating the strains in MH broth at 30°C over a period of time. Crystal violet staining was carried out to quantify separately the biofilm attached to the culture tubes and the pellicle formed at the air-liquid interface. *acrR* mutant exhibited increased biofilm formation even though the growth rate of the mutant was similar to that of wild type (Figure 5). The wild type *A. nosocomialis* displayed maximum biofilm formation at 48 h while the *acrR* mutant at 72 h (Figure 5A). There was a 2-fold increase in biofilm formation in the *acrR* mutant at 72 h compared to that of wild type. In the case of complemented mutant, the biofilm formation was almost similar to that of wild type (Figure 5B). As in the case of biofilm formation, the pellicle formation was also increased in the *acrR* mutant compared to that of wild type at different time periods (48 and 72 h) (Figure 5C). The highest pellicle formation was observed in the *acrR* mutant at 72 h. The above results suggest that AcrR is a negative regulator of biofilm/pellicle formation in *A. nosocomialis*.

### *acrR* deletion enhances cell adhesion and invasion

**Figure 6:**
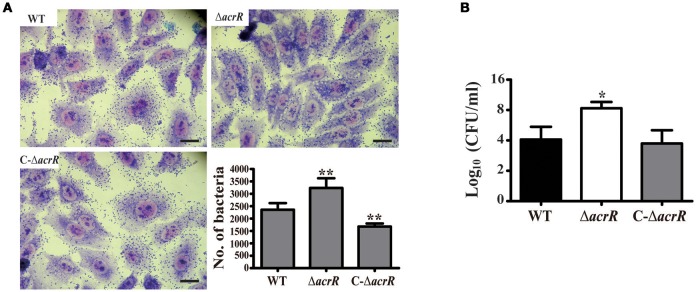
*acrR* deletion mutant displays increased cell adhesion and invasion. To check the effect of *acrR* deletion on the adherence and invasiveness of *A. nosocomialis*, A549 epithelial cells were infected with wild type (WT), *acrR* mutant (Δ*acrR*) and complemented mutant (C-Δ*acrR*) at MOI 100, and incubated at 37°C in 5% CO_2_ for 5 h. **(A)** For the adhesion assay, the infected cells were fixed with methanol post-infection and stained with Giemsa solution. The adherence ability of *acrR* mutant was higher than that of WT and C-Δ*acrR*. The number of bacteria adhered to the epithelial cells is graphically represented. Values are mean ± SD (*n* = *3*) and asterisks indicate significantly different average bacterial count when compared with wild type at **P < 0.01. Scale bar, 50 μm. **(B)** To check the effect of *acrR* deletion on the invasiveness of *A. nosocomialis*, the extracellular bacteria were removed post-infection followed by cell lysis and CFU counting. Values are mean ± SD (*n* = *3*) and asterisk indicate significantly different average CFU value when compared with wild type at *P < 0.05.

Multidrug efflux pumps including AcrAB-TolC efflux system play an important role in the adherence and invasiveness of pathogenic bacteria to the host cells (Hirakata et al., 2002; Buckley et al., 2006). We determined the adherence and invasiveness of *acrR* mutant to A549 epithelial cells and noticed higher adherence and invasion ability compared to the wild type (Figure 6, Supplementary Figure 1). The adherence and invasiveness of the complemented mutant was similar to that of the wild type. The data depicts that AcrR plays a negative role in the adherence and invasiveness of *A. nosocomialis*. We checked the expression level of the genes, *csuC* and *csuD* which encodes components of the CsuA/BABCDE chaperone-usher pili assembly system important for the assembly and production of pili involved in adhesion in *Acinetobacter* by mRNA expression analysis. It was demonstrated that the expression of *csuC* and *csuD* genes are upregulated in the *acrR* deletion mutant compared to the wild type (Supplementary Figure 2). Thus, it can be concluded that the elevated expression of *csuC* and *csuD* might contribute to the increased adhesion and invasion in the *acrR* mutant.

### *acrR* deletion mutant displays increased virulence

**Figure 7:**
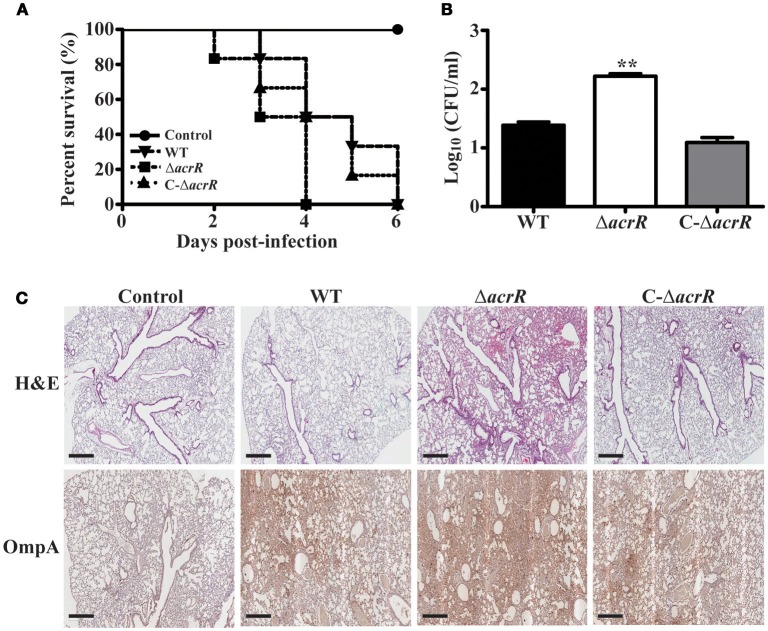
Deletion of *acrR* promotes virulence in *A. nosocomialis*. To investigate the effect of *acrR* deletion on the virulence of *A. nosocomialis*, mice were intratracheally infected with *A. nosocomialis* wild type (WT), *acrR* mutant (Δ*acrR*) and complemented mutant (C-Δ*acrR*). **(A)** Survival curve of mice infected with different bacterial strains. The lethality was higher in the case of mice infected with *acrR* mutant compared to the wild type. **(B)** Bacterial burden in lungs infected with *A. nosocomialis* strains. Lungs were homogenized 3 days post-infection and CFU counting was performed. **(C)** H&E, IHC staining results showing severe tissue damage in lungs of mice infected with *acrR* mutant compared to that with wild type. Values are mean ± SD (*n* = *3*) and asterisks indicate a significant difference in CFU value compared with the wild type at **P < 0.01. Scale bar, 500 μm.

It has been reported previously that the AcrAB-TolC efflux system plays a direct role in the virulence of pathogenic bacteria. In *S*. Typhimurium, *acrB* insertional inactivated/deleted mutant displayed less virulence compared to the wild type strain (Buckley et al., 2006; Perrett et al., 2009; Webber et al., 2009). To investigate the role of AcrR on virulence, we infected BALB/c mice with wild type, *acrR* mutant and complemented mutant of *A. nosocomialis* intratracheally. Survival curve was plotted to assess the overall virulence of the mutant compared to the wild type. As shown in Figure 7A, the mice infected with the *acrR* mutant had a significantly lower survival rate. The CFUs of the mutant in the lungs of infected mice were 1.5 times higher than that of wild type in the infected lungs (Figure 7B). In addition, H&E staining and IHC of the lung tissues revealed that the *acrR* mutant severely damaged the lungs compared to the wild type (Figure 7C). The lung tissues from *acrR* mutant depicted increased cellular infiltration compared to that of the wild type. The IHC data showed higher number of bacterial cells in the damaged lung tissues from the *acrR* mutant than from the wild type tissues. The above results demonstrate that AcrR suppresses virulence in *A. nosocomialis* and thus the *acrR* mutant is highly pathogenic than the wild type.

## Discussion

Multidrug efflux pumps have been recognized as playing a pivotal role in drug resistance, cell division, pathogenicity and biofilm formation (Nikaido, 1996; Buckley et al., 2006; Kvist et al., 2008). Here, we identified an operon encoding AcrA and AcrB in *A. nosocomialis*, which shows significant homology to the AcrAB efflux pump of *E. coli*. The *acrAB* operon in *A. nosocomialis* depicted high similarity to the *arpAB* operon which plays a role in aminoglycoside resistance in *A. baumannii* 5075 (Tipton et al., 2017). In *E. coli*, the *acrAB* operon is regulated by AcrR, encoded by *acrR* located 140 bp upstream of the *acrAB* operon (Ma et al., 1996). The AcrR belongs to TetR family of transcriptional regulators and is composed of an N-terminal domain containing a DNA-binding helix-turn-helix (HTH) motif, and a C-terminal domain containing an unique ligand-binding sequence (Ramos et al., 2005; Li et al., 2007; Su et al., 2007). *In silico* analysis revealed that the DNA-binding motif of AcrR is highly conserved in the bacterial community (data not shown). Similar to that in *E. coli*, the *acrR* in *A. nosocomialis* is located upstream of *acrAB* operon and is divergently transcribed from the same promoter of *acrA*. Also, the AcrR binding motif identified in the P*acrA*/*acrR* of *A. nosocomialis* showed close similarity to that in *E. coli*.

*In silico* analysis and further confirmation by EMSA revealed that AcrR of *A. nosocomialis* binds to the promoter region of *acrA/acrR* and might control the expression of *acrAB*. The expression of *acrAB* was elevated in the *acrR* mutant, which was confirmed by RT-PCR. In *E. coli*, AcrR negatively modulates the expression of the AcrAB efflux pump and the absence of functional AcrR resulted in increased transcription of *acrAB* (Ma et al., 1996). In addition, AcrR acts as an autorepressor regulating its own gene expression in *E. coli*.

It was found that deletion of *acrR* leads to increased motility in *A. nosocomialis*, which is in accordance with the motility observed in the *acrR* mutant of *E. coli* (Kim et al., 2016). In *E. coli*, the deletion of *acrR* induces transcription of most motility genes, indicating that the toxic-compounds-response regulator AcrR participates in motility to escape toxic compounds. The inactivation of *acrB* in *S. enterica* negatively impacts survival in the host and motility (Webber et al., 2009) contributing to reduced pathogenicity (Khoramian-Falsafi et al., 1990). The data from our study reveal that AcrR has a negative role to play in motility by controlling the expression of AcrAB efflux pump.

The absence of the AcrAB efflux pump repressor, AcrR contributed to increased biofilm/pellicle formation, indicating that the AcrAB efflux pump is positively associated with biofilm formation in *A. nosocomialis*. It has been reported previously that a number of multidrug efflux systems, including AcrAB-TolC, play a role in biofilm formation in various Gram negative species, including *E. coli, Klebsiella* and *S*. Typhimurium (Kvist et al., 2008; Baugh et al., 2012). In addition, an upregulated expression of multidrug efflux genes, *acrA* and *acrB* has been noticed in biofilm cells of *E. coli* (Zou et al., 2012). The study that explores the functional link between multidrug efflux pump and biofilm formation in *S*. Typhimurium revealed that several multidrug efflux pumps, including AcrAB plays a role in the formation of extracellular matrix (Baugh et al., 2014). It has been known that the extracellular biofilm matrix of *E. coli* and *Salmonella* is majorly composed of structural curli proteins which help them in adhesion to inert surfaces as well as to host cells (Cegelski et al., 2009; Baugh et al., 2014). The curli proteins are constituted by the major and minor curli subunits which are synthesized by the *csgBA* operon (Grund and Weber, 1988; Andersson et al., 2013). It has been noticed previously that the *acrB* and *tolC* mutants of *S*. Typhimurium lack the production of curli due to the transcriptional repression of *csgA* and *csgB* genes (Baugh et al., 2014), indicating the importance of AcrAB efflux pump in curli production.

It has been known for decades that there is a significant correlation between biofilm formation and multidrug resistance (Anderl et al., 2000). The bacterial cells which are associated to biofilm can tolerate antibacterial agents far better than planktonic cells. The MexAB-OprM and MexCD-OprJ pumps of *P. aeruginosa* have been shown to be involved in biofilm-specific mechanisms for resistance (Gillis et al., 2005). In addition, the efflux pump operon, PA1874-1877 in *P. aeruginosa* was seen upregulated during biofilm formation and that appeared to be involved in biofilm-mediated antibiotic resistance (Zhang and Mah, 2008). Further studies on the expression level of AcrAB efflux pump and multidrug resistance under biofilm-forming conditions would shed light on the involvement of this pump in biofilm-mediated antibiotic resistance in *A. nosocomialis*.

The invasiveness and pathogenesis of *A. nosocomialis* was elevated when the *acrR* gene was deleted. Earlier studies have shown that the AcrAB-TolC efflux system and its homologs are crucial for the invasion/virulence of *S*. Typhimurium, *Moraxella catarrhalis, P. aeruginosa*, and *Erwinia amylovora* (Hirakata et al., 2002; Burse et al., 2004; Spaniol et al., 2015; Wang-Kan et al., 2017). Buckley et al. reported that functional AcrB is important for adhesion, invasion and survival of *S*. Typhimurium in eukaryotic cells, and the impaired virulence in the AcrB-defective mutant is due to the loss of efflux pump activity (Buckley et al., 2006). It was suggested that the poor invasion/intracellular survival of the *acrB* mutant might be accounted by the inability of the bacteria to export host antimicrobials, such as basic peptides or toxic compounds present in lysosomes as the natural function of the AcrAB-TolC efflux system is to expel such compounds from the bacterial cell (Shafer et al., 1998; Buckley et al., 2006). In addition, the decrease in adhesion and/or invasion of the *acrB* mutant could also be due to the lack of secretion of adhesins, which might be substrates of AcrAB-TolC efflux pump (Buckley et al., 2006). Furthermore, transcriptome analysis of the *acrB* mutant depicted downregulation of *Salmonella* pathogenicity island (SPI) genes that are essential for invasion, bacterial survival and persistence within the host (Webber et al., 2009). Also, it is known that multidrug efflux systems are required for the optimal production of virulence factors besides its role in the export of toxic chemicals (Burse et al., 2004; Bina et al., 2008). In this study, the deletion of *acrR* led to the upregulation of *csuC* and *csuD* genes which are important for the production and assembly of the virulence factor pili needed for adhesion in *A. nosocomialis*. Based on the fact that *acrAB* operon is induced in the *acrR* mutant, it could be suggested that the overexpression of the AcrAB efflux pump might play a role in the enhanced production of virulence factors contributing to increased biofilm formation, adhesion, invasion and pathogenesis in *A. nosocomialis*.

The involvement of multidrug efflux pumps in quorum sensing (QS) was first reported in *P. aeruginosa* in which the MexAB-OprM plays a role in the efflux of autoinducer N-(3-oxododecanoyl)-L-homoserine lactone (3-oxo-C12-HSL) (Pearson et al., 1999). The *mexAB*-*oprM* mutant displayed reduced diffusion of the 3-oxo-C12-HSL into the extracellular environment resulting in loss of virulence. The transcription of quorum sensing transporter genes *lsrACDBK* and the operon repressor *lsrR* was decreased in the *acrB* mutant of *S*. Typhimurium (Wang-Kan et al., 2017), indicating the role of AcrAB in the expression of virulence factors as *lsrR* controls the expression of SPI-related genes (Choi et al., 2012). In *Burkholderia pseudomallei*, the extracellular secretion of AHLs is absolutely dependent on the function of BpeAB-OprB efflux pump (Chan et al., 2007). Interestingly, in our study, RNA sequencing and mRNA expression analysis of the *anoR* mutant of *A. nosocomialis* depicted the downregulation of *acrA* and *acrB* genes (data unpublished). It has been reported earlier that the expression of *mexAB-oprM* of *P. aeruginosa* is limited by the intracellular concentration of the autoinducer molecules (Maseda et al., 2004). Thus, the decreased expression of *acrA* and *acrB* in the *anoR* mutant could be very well-associated with the absence of AHLs in the mutant background. It is very well-known that QS plays a crucial role in motility, biofilm formation and the production of virulence factors (Rutherford and Bassler, 2012). Thus, the enhanced AcrAB efflux pump activity in the *acrR* mutant might attribute well to the AHLs-mediated cell-to-cell signaling, contributing to increased motility, biofilm formation and virulence in the mutant.

In summary, we report a novel regulator, AcrR which negatively regulates the uncharacterized multidrug efflux operon, *acrAB* in *A. nosocomialis*. The bacterial motility, biofilm/pellicle formation and virulence were increased in the *acrR* mutant with the upregulated expression of *acrAB* operon. This supports the notion that besides contributing to antibiotic resistance, multidrug efflux pumps display a variety of functions in accordance to the environment and the bacterial behavior. Further studies are ongoing for in-depth characterization of the AcrAB efflux pump so that the knowledge could be used for developing efflux pump inhibitors as they are invaluable tools to restore the activity of agents to which the efflux pump confers resistance and also to reduce the ability of bacteria to infect their host.

## Author contributions

BS, JK, MO, and CC designed the research. BS, JK, DK, KW, and MO performed the research. BS, JK, DK, KW, MO, and CC analyzed the data. BS, MO, and CC wrote the paper.

### Conflict of interest statement

The authors declare that the research was conducted in the absence of any commercial or financial relationships that could be construed as a potential conflict of interest.
