# Supplementary material for: Local Repressor AcrR Regulates AcrAB Efflux Pump Required for Biofilm Formation and Virulence in Acinetobacter nosocomialis
Source: Front Cell Infect Microbiol. 2018 Aug 7;8:270. doi: 10.3389/fcimb.2018.00270 (PMC6090078; doi:10.3389/fcimb.2018.00270)
Supplement: Supplementary file 1 [file Table_1.docx]

Supplementary Material

**Local Repressor AcrR Regulates AcrAB Efflux Pump Required for Biofilm formation and Virulence in *Acinetobacter nosocomialis***

Bindu Subhadra^1^, Jaeseok Kim^1^, Dong Ho Kim^1^, Kyungho Woo^1^, Man Hwan Oh^2*^, Chul Hee Choi^1*^

^1^Department of Microbiology and Medical Science, Chungnam National University School of Medicine, Daejeon, South Korea

^2^Department of Nanobiomedical Science, Dankook University, Cheonan, South Korea

*** Correspondence:**

Man Hwan Oh: [yy1091@dankook.ac.kr](mailto:yy1091@dankook.ac.kr)

Chul Hee Choi: [choich@cnu.ac.kr](mailto:choich@cnu.ac.kr)

**
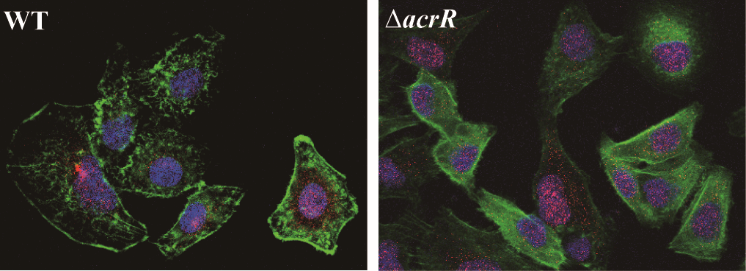
**

**Supplementary Figure 1.** Fluorescence microscopy displayed increased invasion of *acrR* deletion mutant. To visualize the invaded bacteria by fluorescence microscopy, A549 epithelial cells seeded on glass coverslips were stained with polyclonal anti-rabbit AbOmpA antibody, followed by Alexa Fluor^®^ 594-conjugated goat anti-rabbit IgG antibody (red) post-infection. Actin filaments and nuclei were stained with Alexa Fluor^®^ 488 phalloidin (green) and DAPI (blue) respectively. The merged images are shown.
