# Supplementary material for: Local Repressor AcrR Regulates AcrAB Efflux Pump Required for Biofilm Formation and Virulence in Acinetobacter nosocomialis
Source: Front Cell Infect Microbiol. 2018 Aug 7;8:270. doi: 10.3389/fcimb.2018.00270 (PMC6090078; doi:10.3389/fcimb.2018.00270)
Supplement: Supplementary file 2 [file Table_2.docx]

Supplementary Material

**Local Repressor AcrR Regulates AcrAB Efflux Pump Required for Biofilm formation and Virulence in *Acinetobacter nosocomialis***

Bindu Subhadra^1^, Jaeseok Kim^1^, Dong Ho Kim^1^, Kyungho Woo^1^, Man Hwan Oh^2*^, Chul Hee Choi^1*^

^1^Department of Microbiology and Medical Science, Chungnam National University School of Medicine, Daejeon, South Korea

^2^Department of Nanobiomedical Science, Dankook University, Cheonan, South Korea

*** Correspondence:**

Man Hwan Oh: [yy1091@dankook.ac.kr](mailto:yy1091@dankook.ac.kr)

Chul Hee Choi: [choich@cnu.ac.kr](mailto:choich@cnu.ac.kr)


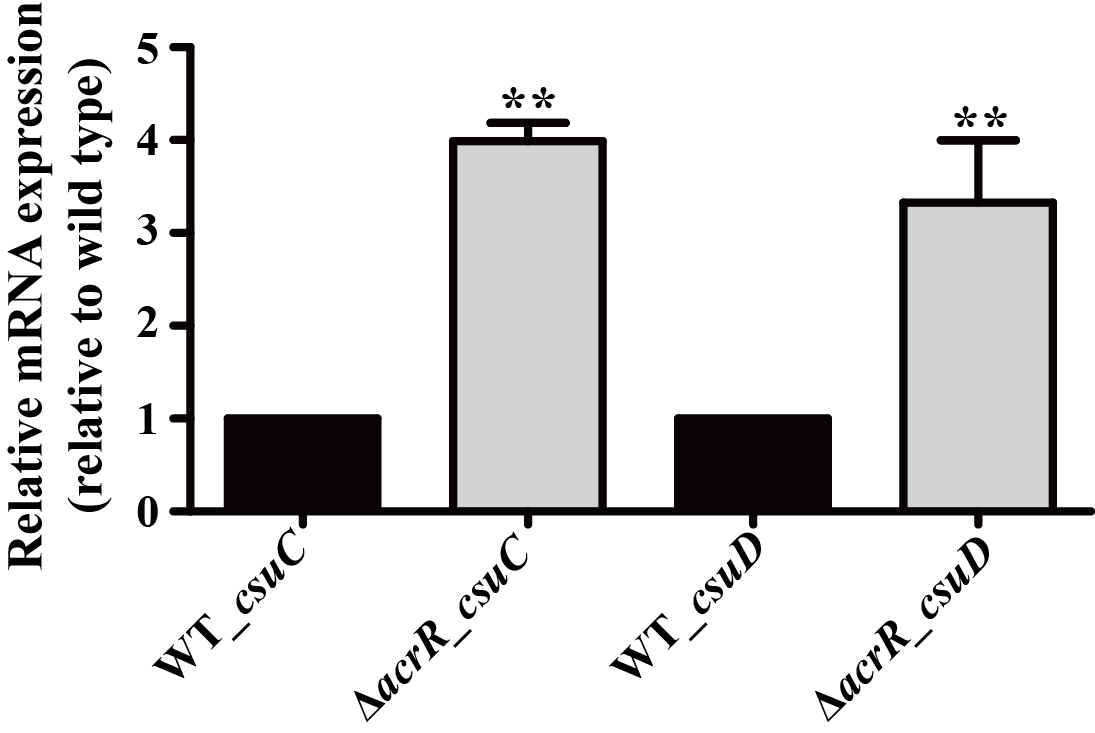


**Supplementary Figure 2.** Transcription of *csuC* and *csuD* genes are upregulated in the *acrR* deletion mutant. The protocol followed for measuring the relative mRNA expression level in the wild type (WT) and *acrR* mutant (∆*acrR*) is given in the ‘Materials and Methods’ section. Values are mean ± SD (*n=3*) and asterisks indicate a significant difference in mRNA expression between the mutant and the wild type at ***P* < 0.01.
