# Supplementary material for: Local Repressor AcrR Regulates AcrAB Efflux Pump Required for Biofilm Formation and Virulence in Acinetobacter nosocomialis
Source: Front Cell Infect Microbiol. 2018 Aug 7;8:270. doi: 10.3389/fcimb.2018.00270 (PMC6090078; doi:10.3389/fcimb.2018.00270)
Supplement: Supplementary file 3 [file Table_3.docx]

Supplementary Material

**Local Repressor AcrR Regulates AcrAB Efflux Pump Required for Biofilm formation and Virulence in *Acinetobacter nosocomialis***

Bindu Subhadra^1^, Jaeseok Kim^1^, Dong Ho Kim^1^, Kyungho Woo^1^, Man Hwan Oh^2*^, Chul Hee Choi^1*^

^1^Department of Microbiology and Medical Science, Chungnam National University School of Medicine, Daejeon, South Korea

^2^Department of Nanobiomedical Science, Dankook University, Cheonan, South Korea

*** Correspondence:**

Man Hwan Oh: [yy1091@dankook.ac.kr](mailto:yy1091@dankook.ac.kr)

Chul Hee Choi: [choich@cnu.ac.kr](mailto:choich@cnu.ac.kr)

# Supplementary Table 1. Bacterial strains used for the construction of phylogenetic tree.

| **Strains** | **GenBank accession no.** | **Locus tag** |
| --- | --- | --- |
| *A. nosocomialis* ATCC 17903 | CP029351 | DIW83_00165 |
| *A. nosocomialis* 6411 | NZ_CP010368 | RR32_RS00160 |
| *A. baumannii* 1656-2 | NC_017162 | ABK1_RS18815 |
| *A. baumannii* ATCC 17978 | NC_009085 | A1S_3448 |
| *A. baylyi* ADPI | NC_005966 | ACIAD_RS16395 |
| *Mannheimia haemolytica PHL213* | NZ_DS264610 | MHA_RS10590 |
| *Vibrio parahaemolyticus RIMD 2210633* | NC_004603 | VP0040 |
| *Ferrimonas balearica* DSM 9799 | NC_014541 | FBAL_RS13770 |
| *Micrococcus luteus* NCTC 2665 | NC_012803 | MLUT_RS22600 |
| *Pseudomonas aeruginosa* DSM 50071 | CP012001 | PA50071_06375 |
| *Pseudomonas putida* NBRC 14164 | NC_021505 | PP4_RS21150 |
| *Pseudomonas protegens* Pf-5 | NC_004129 | PFL_RS05860 |
| *Pseudomonas syringae* KCTC 12500 | NZ_AYTM02000002 | V476_RS01805 |
| *Tepidiphilus margaritifer* DSM 15129 | NZ_AUDR01000013 | H607_RS11245 |
| *Roseovarius* sp. 217 | NZ_CH902584 | ROS217_RS07325 |
| *Ralstonia solanacearum* CFBP2957 | FP885897 | RCFBP_20785 |
| *Burkholderia cepacia* LMG 16656 | NZ_JTDP01000001 | NP88_RS03690 |
| *Enterobacter cloacae* subsp. *cloacae* ATCC 13047 | NC_014121 | ECL_01235 |
| *Yokenella regensburgei* ATCC 49455 | NZ_JMPS01000025 | GYRE_RS07890 |
| *Klebsiella pneumoniae* ATCC 13883 | NZ_KN046818 | DR88_RS12595 |
| *Salmonella enterica* subsp. *enterica* serovar  Typhimurium LT2 | NC_003197 | STM0477 |
| *E. coli* K-12 substr. MG1655 | NC_000913 | b0464 |
